# VR preoperative education with sensory information alleviates perioperative anxiety and enhances delivery experience in primiparas undergoing cesarean section: a randomized controlled trial

**DOI:** 10.3389/fpsyg.2025.1648706

**Published:** 2026-05-20

**Authors:** Xu Ran, Yanlin Wei, Rong Huang, Chengxi Tang, Yanhua Hu, Diqiao Wang

**Affiliations:** 1 The Second Clinical Medical College of North Sichuan Medical College, Nanchong, China; 2 Beijing Anzhen Nanchong Hospital, Capital Medical University and Nanchong Central Hospital, Nanchong, China

**Keywords:** anxiety, cesarean section, delivery experience, sensory information, virtual reality

## Abstract

**Purpose:**

To investigate the effectiveness of a VR preoperative education program with sensory information support in reducing perioperative anxiety and improving the delivery experience among primiparas undergoing cesarean section (CS).

**Methods:**

A total of 108 primiparas undergoing elective cesarean section were recruited and randomly assigned to either the VR group or the Control group. The control group received routine preoperative education, whereas the VR group also received a sensory information-based VR intervention 1 day before surgery. The primary outcomes were anxiety levels at the preoperative, intraoperative, and post-operative stages, as well as the delivery experience. The secondary outcomes were preoperative readiness, satisfaction, post-operative recovery, as well as vital signs (mean arterial pressure and heart rate). Repeated measures analysis of variance was used to compare differences in perioperative anxiety, mean arterial pressure, and heart rate between the two groups. Independent samples *t*-tests were used to compare preoperative readiness, delivery experience, and satisfaction between the two groups.

**Results:**

A significant group-by-time interaction was observed for anxiety (*P* < 0.05). *Post-hoc* simple effects analysis showed that preoperative anxiety was significantly lower in the VR group compared to the control group (*P* < 0.05). Within-group analyses revealed that anxiety levels remained stable in the VR group, whereas they increased significantly from baseline at both the preoperative and intraoperative stages in the control group (*P* < 0.05). A significant group-by-time interaction was observed for MAP (*P* < 0.05). *Post-hoc* analysis showed that the VR group had significantly lower MAP than the control group upon entering the operating room and before anesthesia (*P* < 0.05). Within-group comparisons revealed that the VR group's MAP was elevated from baseline only before anesthesia, whereas the control group showed significant elevations both before anesthesia and upon entering the operating room (*P* < 0.05). A significant main effect of time was found for HR (*P* < 0.05), with no significant main effect of group or time-by-group interaction. The delivery experience, preoperative readiness, and satisfaction were higher in the VR group than those in the control group (*P* < 0.05), and there was no significant difference in post-operative recovery (*P* > 0.05).

**Conclusion:**

The VR-based sensory information support intervention effectively alleviated preoperative anxiety, enhanced the delivery experience, improved preoperative readiness and satisfaction, and stabilized preoperative blood pressure in primiparas undergoing cesarean section.

## Introduction

Cesarean section (CS) is a surgical procedure involving an abdominal incision into the uterus to deliver the fetus and placenta. It is a crucial intervention for reducing maternal and infant mortality Ellis. According to WHO statistics, the global CS rate was 21% in 2021 and is projected to rise to 30% by 2030. In China, the average CS rate was notably higher, recorded at 43.79 and 46.5% in 2020 and 2021, respectively (Yin et al., 2023).

CS is a significant physiological and psychological stressor that can negatively impact the maternal delivery experience. The prevalence of preoperative anxiety among parturients undergoing CS is as high as 60%−80%, primarily due to concerns for their own and their fetus's safety (Eslami et al., 2020). Furthermore, as neuraxial anesthesia is the preferred technique for CS, parturients remain awake with intact sensory function during the procedure. This heightened awareness renders them more susceptible to the OT environment, predisposing them to develop negative emotions such as anxiety and fear (Vusqa et al., 2019). Excessive anxiety not only creates a negative delivery experience but can also induce hypertension, tachycardia, and increase the risk of hemorrhage. It is also associated with a higher prevalence of postpartum depression, prolonged recovery, and can lead to delays in or avoidance of future pregnancies (Halla et al., 2020; Tonei, 2019; Benton et al., 2019).

There are two primary approaches to managing perioperative anxiety: pharmacological and non-pharmacological. parturients are not recommended to use sedatives for considering maternal and neonatal safety Wilson. Non-pharmacological methods such as music therapy and prenatal education can reduce maternal perioperative anxiety (Kirdemir et al., 2024; Endalew et al., 2022) music interventions may require interventionists to receive adequate professional training to develop tailored music experiences, which may be limited in some health care settings (Drzymalski et al., 2023). Whereas, the ideal method for presenting information in antenatal education is unclear, written information is the main method of providing information and education to maternity, but not all parturients have sufficient reading skills to understand the information. Multimedia information methods such as video instruction are also used in maternal education, but a systematic review showed inconsistency in the effectiveness of video education in reducing perioperative anxiety, which needs to be further explored (Maghalian et al., 2024). Most importantly, in today's era of rapid technological advancement, traditional and non-interactive interventions may not be able to meet the needs and expectations of parturients. Given the limitations of previously used non-pharmacological interventions, the development of new and advanced technologies, such as VR, offers new directions for managing perioperative anxiety.

Virtual reality (VR) utilizes computer graphics and simulation technologies to create immersive environments that integrate realistic visual, auditory, and tactile stimuli, evoking the sensation of being in a real-world setting. VR has demonstrated efficacy in managing perioperative anxiety in adults (Ince and Karaman, 2024; Steinkraus et al., 2024; Xu, H et al., 2024). In obstetrics, the intraoperative application of VR has been shown to reduce maternal anxiety and improve satisfaction. However, its effectiveness in reducing preoperative anxiety remains controversial. While one scoping review indicates that VR can be beneficially applied throughout pregnancy—from alleviating anxiety and pain to facilitating exercise training and enhancing the understanding of childbirth (Hajesmaeel-Gohari et al., 2021)—other studies have found no statistically significant anxiolytic effects. This inconsistency may be attributed to heterogeneity in VR content and intervention design, which underscores the need for standardized, evidence-based approaches tailored to the psychophysiological needs of pregnant women. Notably, recent interventional studies conducted during pregnancy and labor further indicate that interventions integrating immersive technologies with breathing-based techniques may yield more consistent and clinically meaningful benefits. In this context, (Karacan and Akkoz Cevik 2024) demonstrated that diaphragmatic breathing exercises were more effective than virtual reality applications in reducing labor pain and duration and in enhancing maternal satisfaction during the latent phase of labor. These findings underscore the importance of autonomic regulation and embodied self-control mechanisms in perinatal anxiety and pain management, beyond the immersive qualities of VR alone.

The therapeutic mechanism of VR warrants clarification. VR interventions exert their effects primarily by immersing individuals in controlled, multisensory environments that can influence cognitive appraisal, emotional regulation, and perceived control over upcoming experiences. In the context of medical procedures, the therapeutic potential of VR is therefore not limited to distraction alone but extends to its capacity to systematically structure and deliver sensory information in an anticipatory manner. Sensory information involves preparing patients for the physical sensations they may experience during medical procedures by describing the visual, auditory, and tactile stimuli associated with the event (Freeman et al., 2018). Building on this premise, we integrated structured sensory information into a VR-based intervention and conducted a randomized controlled trial to investigate whether such preoperative education could reduce perioperative anxiety and improve the childbirth experience in women undergoing cesarean section.

## Methods

### Preliminary work

In accordance with the recommendations of the International Working Group on Methodological Design of Virtual Reality Clinical Trials in Healthcare Birckhead, we conducted three phases (VR1, VR2, and VR3) of clinical trials to ensure the scientificity of VR development.

In the VR1 phase, we first structured the educational content around a sensory information protocol. This protocol was designed to mitigate anxiety through three mechanisms: providing procedural details to reduce uncertainty, describing typical sensations to prepare for the experience, and offering behavioral guidance for managing adverse sensations. This content was then produced into a 6.6-min VR video from the first-person perspective of a primipara. The video comprised three segments: ①preoperative preparations, ②a detailed walkthrough of the surgical pathway which explicitly described potential discomforts and provided coping strategies, ③post-operative analgesia education. To minimize distress, all invasive and graphic imagery was omitted, and a supportive voiceover narrated the process, emphasizing the controllable nature of any discomfort.

In the VR2 phase, a pilot trial was conducted with a sample size of 20 subjects who were randomly assigned to either the VR group or the control group (allocation ratio of 1:1), and the trial demonstrated that VR could be applied to primiparas with high acceptance.

In the VR3 phase, a randomized controlled trial (this study) was conducted to evaluate the clinical outcomes of the VR intervention in primiparas undergoing CS.

### Study design

This study was a prospective, randomized controlled trial from March 2024 to November 2024, approved by the Ethics Committee of Nanchong Central Hospital ([2024]023). Informed written consent was obtained from all subjects, emphasizing their right to withdraw under any circumstances.

### Participants and setting

The study was conducted in the obstetrics department of a tertiary hospital in Nanchong, China. Primiparas were invited to participate in the study if they met all of the following criteria: ① CS with elective neuraxial anesthesia and anesthesia ASA grade I-II, ② primipars' age ≥20 and gestational age ≥37 weeks, ③ single fetus pregnancy and no fetal abnormality in antenatal examination, ④ normal vision and hearing, ⑤ consent to participate in the study. Primiparas were excluded if they met any of the following conditions: ① serious pregnancy complications, such as intrauterine distress, placental abruption, uterine rupture, severe eclampsia, severe hyperemesis gravidarum and other critical illnesses, ② the presence of generalized anxiety disorder or history of psychiatric disorders, ③ history of vestibular dysfunction. Primiparas were withdrawn from the study if any of the following occurred: ① conversion from neuraxial to general anesthesia, ② life-threatening intraoperative complications requiring emergency resuscitation, such as major hemorrhage (estimated blood loss >1,000 ml), total spinal anesthesia, or amniotic fluid embolism, ③ Delivery of a neonate with an Apgar score of ≤ 7 at 5 min requiring admission to the neonatal intensive care unit, ④ requests for withdrawal from the study.

### Sample size

The sample size was calculated based on a meta-analysis of 10 randomized controlled trials (Xu, H et al., 2024), which reported an effect size of 0.64 for VR interventions on anxiety reduction. Using G^*^Power software with a statistical power of 80% and a two-sided alpha level of 0.05, a minimum of 45 participants per group was required. Accounting for an anticipated dropout rate of 20%, the total target sample size was set at 108 participants (54 per group).

### Randomization and blinding

Participants were randomly assigned to either the VR group or the control group. The random allocation sequence was generated by a research assistant not involved in the study using Excel software with a 1:1 allocation ratio. The assignments were concealed in sequentially numbered, opaque, sealed envelopes. On the day of admission, participants opened the envelopes to reveal their group assignment. This process ensured that only the participants and the interventionist were aware of the group allocation, while the operating surgeons, anesthesiologists, and other clinical staff remained blinded to the group assignment.

### Intervention content

The control group received standard care, which included: ①preoperative education: instructions on diet, hygiene, and procedure from ward nurses, an OR nurse, and an anesthesiologist during preoperative visits, ②transfer to OR: Identity verification and transfer from the ward to the operating room by nursing staff, ③preoperative verification: a final identity check in the OR holding area and the operating room itself, ④intraoperative protocol: standard monitoring, IV access, neuraxial anesthesia, urinary catheter insertion, disinfection and surgical procedures, ⑤post-operative transfer and care: return to the ward followed by routine vital signs monitoring and post-operative instructions.

In addition to standard care, participants in the VR group received a supplementary VR video intervention. The video, structured around 15 distinct scenes across three primary locations, delivered health education content underpinned by sensory information principles. The first segment covered preoperative preparations (e.g., diet, attire, personal items). The second segment detailed the surgical pathway from the ward to the operating room and back. Crucially, this section explicitly prepared primiparas for potential discomforts and provided specific coping strategies. The voiceover employed direct and reassuring language to describe anticipated sensations and outline management techniques. For example, it addressed tension during the preoperative period by suggesting the use of available resources, such as a tablet device or structured communication with staff. Regarding neuraxial anesthesia, it explained the sequence of a brief prick, warmth, and numbness as normal, with instructions provided to maintain position, breathe deeply, and report radiating pain. For urinary catheterization, the narration clarified that while pain should be absent, a sensation of fullness might occur, primiparas were guided to use imagery for muscle relaxation, with the discomfort typically subsiding quickly. To address potential nausea from visceral traction, primiparas were instructed to perform controlled breathing and maintain a specific head position under supervision. The narration also noted that antiemetic medication was available per protocol if required. The third segment provided post-operative analgesia education, including the use of a patient-controlled analgesia pump.

The VR video was delivered using a Pico Dream Pro head-mounted display, which featured a 93 field of view, a binocular resolution of 3664 × 1920, a 90 Hz refresh rate, and an interpupillary distance adjustment range of 57–69 mm, along with integrated speakers and a microphone. On the day before surgery, an operating room nurse initiated the VR session for the primiparas in a dedicated obstetrics study room. The video content, with a total duration of 6.6 min, was controlled remotely via a tablet computer, ensuring the nurse and the participant viewed synchronized content. Participants were permitted to watch the video once, with the option to repeat it based on their preference. Following the session, the nurse was available to address any questions. The intervention was immediately paused if any participant reported physical or psychological discomfort, with appropriate medical and psychological support provided. The decision to continue with the trial was then made based on the participant's recovered condition and personal willingness. Participants were requested not to share the specific details of the VR intervention with others. All VR equipment was inspected and cleaned with sterile alcohol wipes after each use.

### Intervention fidelity

To ensure consistent delivery of the intervention, all research personnel received standardized training on the operation of the head-mounted VR equipment. This training covered setup procedures, adjustment of the interpupillary distance, and troubleshooting of common technical issues. Furthermore, a detailed, step-by-step protocol was established for the VR intervention to guarantee strict adherence to the study procedures by all nursing staff involved.

### Outcome measures

#### Primary outcomes: perioperative anxiety and delivery experience

The State Anxiety Inventory (SAI) was used to assess anxiety levels in primiparas at four time points: pre-intervention (T0, baseline), on the morning of surgery (T1, pre-surgery), 24 h post-surgery (T2, retrospective intraoperative assessment), and 48 h post-surgery (T3, post-surgery). The retrospective assessment at T2 was implemented because our preliminary study found that primiparas were often fatigued and less cooperative in the operating room immediately after cesarean delivery, making immediate assessment impractical. The SAI measures state anxiety, a transient emotional state characterized by feelings of apprehension, tension, and nervousness. It is a validated instrument for quantifying situational anxiety, with higher scores indicating greater severity.

The delivery experience was evaluated with the Chinese version of the Wijma Delivery Experience Questionnaire (W-DEQ), a 33-item instrument adapted by Liu and Liu (2015) from the original by Wijma. It showed high internal consistency (Cronbach's α = 0.921). Per the scoring guidelines, higher scores reflect a poorer experience, with scores ≥85 indicating a negative childbirth experience that may warrant clinical attention. All primiparas completed the questionnaire within 24 h postpartum.

#### Secondary outcomes

Preoperative readiness for elective surgery: preoperative readiness was assessed using a scale compiled by Guo et al. (2022) 24-item scale, which covers the knowledge, emotional, and behavioral dimensions, demonstrated excellent validity and reliability, with a content validity index of 0.992 and a Cronbach's α of 0.907. Higher total scores are indicative of better preoperative preparedness. Surgical readiness was assessed on the morning of surgery.

Obstetric Quality-of Recovery: post-operative recovery was evaluated using the Obstetric Quality of Recovery Score (ObsQoR-11). This instrument comprises 11 items, each rated on an 11-point scale, resulting in a total score range of 0–110. According to the scoring protocol, higher scores represent a superior quality of recovery (Ffrench-O'Carroll et al., 2024). Recovery was assessed on the third post-operative day.

Maternal satisfaction: the maternal satisfaction scale for cesarean section (MSSCS) was used to assess the maternal satisfaction of cesarean section, which consisted of 22 items, with higher scores indicating higher satisfaction, and the Cronbach' s alpha coefficient was 0.82 Morgan. The questionnaire was completed at 48 h post-surgery.

MAP and HR: SBP, DBP, and HR were monitored using a cardiac monitor. These parameters were recorded in both groups at the following time points: before the intervention, at 7:00 a.m. on the day of surgery, upon entering the OT and before anesthesia. MAP was calculated using the standard formula: MAP = DBP + (SBP – DBP)/3.

Simulation sickness: the Simulator Sickness Questionnaire (SSQ) was used to assess cybersickness symptoms in primiparas of the VR group before and after the intervention. The SSQ Jang is a widely adopted and extensively validated instrument for quantifying simulator-induced discomfort.

To promote complete participation and data collection integrity, all participants received a complimentary post-operative wound dressing change upon completing the study assessments, as well as a small gift for their newborn.

### Statistical analysis

Statistical analyses were performed using SPSS (version 25.0). Continuous variables with a normal distribution are presented as mean ± standard deviation (SD), while non-normally distributed variables are summarized as median and interquartile range. The homogeneity of baseline characteristics between the two groups was compared using the χ^2^ test or Fisher's exact test for categorical variables, and the independent-samples *t*-test or Mann–Whitney *U* test for continuous variables. Outcomes such as anxiety scores, blood pressure, and heart rate were analyzed using repeated-measures ANOVA, with *post-hoc* comparisons between the VR and control groups at each time point adjusted by the Bonferroni method. For other outcome measures (delivery experience, preoperative readiness, satisfaction, and post-operative recovery), between-group comparisons were conducted using the independent-samples *t*-test. A two-sided *P*-value of < 0.05 was considered statistically significant.

## Results

### Characteristics of participants

**Figure 1:**
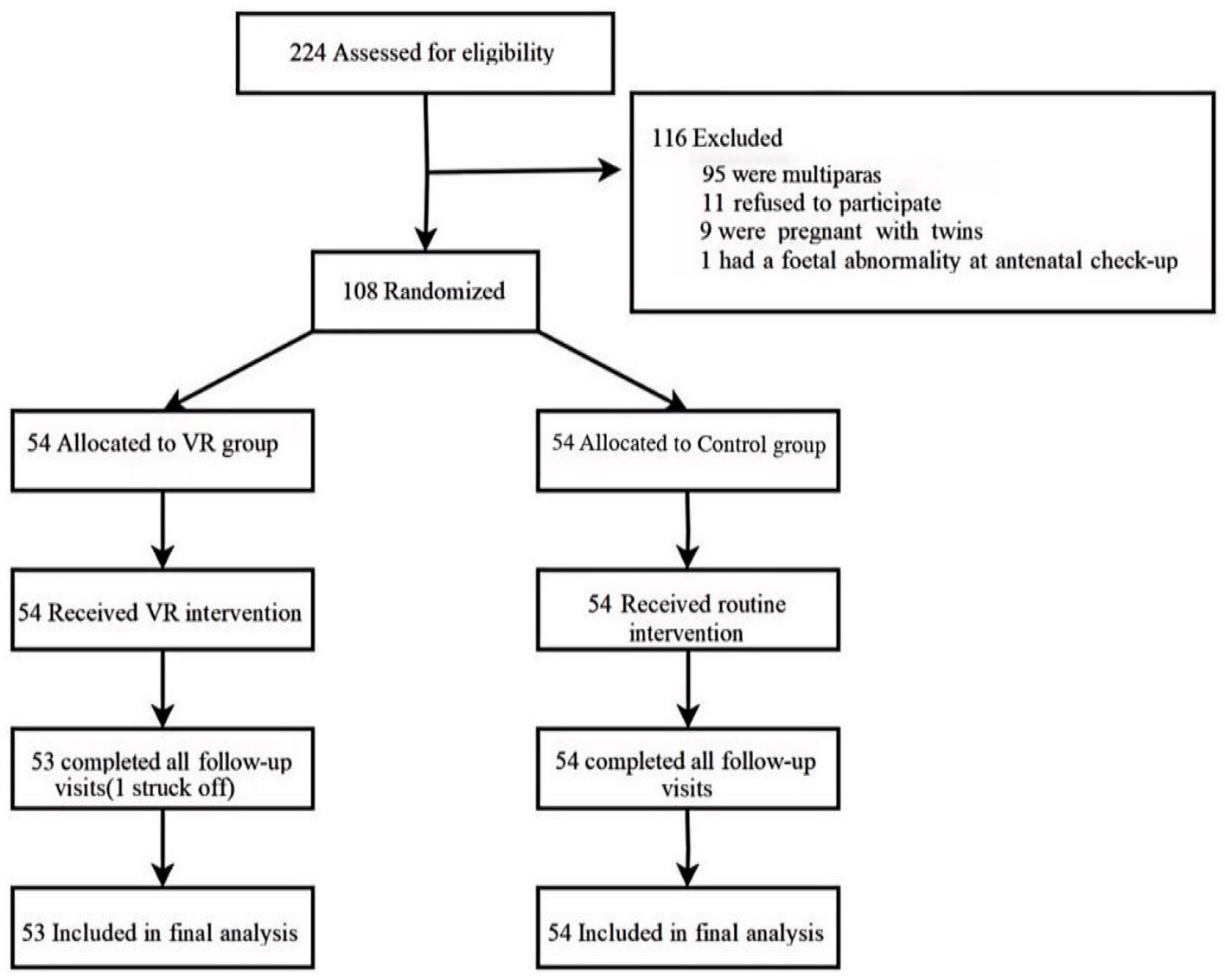
Primiparas enrollment flowchart.

A total of 224 primiparas were assessed for eligibility. Of these, 108 were enrolled and randomly assigned to groups. One participant in the VR group was excluded due to conversion to general anesthesia. Consequently, 107 women completed the intervention and were included in the final analysis (Figure 1). No statistically significant differences in baseline characteristics were observed between the two groups for the primiparas or their newborns (Table 1).

**Table 1:** Subject characteristics.

Characteristics	VR group (*n* = 53)$\bar{\text{x}}$ ±*S*/n (%)	Control group (*n* = 54) $\bar{\text{x}}$ ±*S*/n (%)	*t*/*χ^2^*	*p-*value
Age (year)	27.89 ± 2.79	28.61 ± 3.03	−1.287	0.201
BMI (kg/m^2^)	27.46 ± 3.80	28.01 ± 3.69	−0.756	0.451
Singleton			0.21	0.885
Yes	16 (30.2)	17 (31.5)	
No	37 (69.8)	37 (68.5)	
Ethnicity			0.23	0.632
Han Chinese	50 (94.3)	52 (96.3)	
Various minorities	3 (5.7)	2 (3.7)	
Religious affiliation			0.324	0.569
Yes	1 (1.9)	2 (3.7)	
No	52 (98.1)	52 (96.3)	
Educational level			1.124	0.289
Secondary school or below	25 (47.2)	31 (57.4)	
University or above	28 (52.8)	23 (42.6)	
Profession			6.118	0.295
Workers	11 (20.8)	17 (31.5)	
Farmer	0 (0)	1 (1.9)	
Commercial/service businesses	7 (13.2)	5 (9.3)	
Civil servants	19 (35.8)	11 (20.4)	
Unemployed/job seekers	9 (17)	8 (14.8)	
Others	7 (13.2)	12 (22.2)	
Per capita monthly family income			0.972	0.808
< 3,000 RMB	5 (9.4)	3 (5.6)	
3,000–5,000 RMB	23 (43.4)	22 (40.7)	
5,001–8,000 RMB	16 (30.2)	17 (31.5)	
>8,000 RMB	9 (17)	12 (22.2)	
Medical insurance			0.291	0.590
Workers' medical insurance	36 (67.9)	34 (63)	
Resident medical insurance	17 (32.1)	20 (37)	
Pregnancy planning			0.34	0.56
	44 (83)	47 (87)	
Unplanned	9 (17)	7 (13)	
Type of pregnancy			0.134	0.715
Natural pregnancy	50 (94.3)	50 (92.6)	
Assisted reproductive technology pregnancies	3 (5.7)	4 (7.4)	
Gravidity			0.774	0.679
1	37 (69.8)	40 (74.1)	
2	12 (22.6)	12 (22.2)	
≥3	4 (7.5)	2 (3.7)	
Weeks of pregnancy			1.655	0.647
37^+^	3 (5.7)	3 (5.6)	
38^+^	16 (30.2)	19 (35.2)	
39^+^	26 (49.1)	28 (51.9)	
40^+^	8 (15.1)	4 (7.4)	
Pregnancy complications			0.101	0.751
Yes	8 (15.1)	7 (13)	
No	45 (84.9)	47 (87)	
Duration of anesthesia monitoring (min)	80 (70, 90)	85 (75, 95)	−1.456	0.145
Intraoperative fluid volume (ml)	1,400 (1,100, 1,400)	1,275 (1,200, 1,400)	−0.897	0.37
Intraoperative urine output (ml)	200 (200, 200)	200 (100, 200)	−0.709	0.478
Newborn gender			3.447	0.063
Boys	19 (35.8)	29 (53.7)	
Girls	34 (64.2)	25 (46.3)	
Newborn weight (g)	3,290.47 ± 343.32	3,351.57 ± 347.96	−0.914	0.363
Apgar score (1 min)	10 (10, 10)	10 (10, 10)	−0.679	0.497

### Primary outcomes

**Figure 2:**
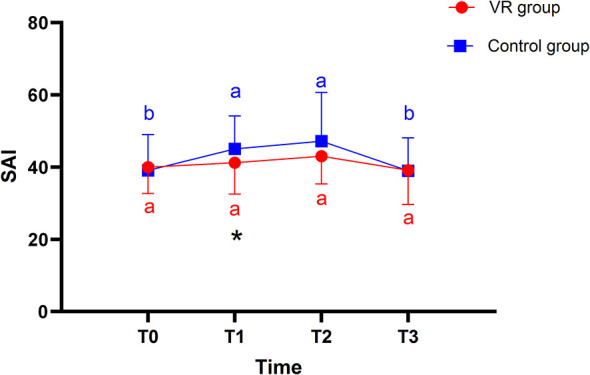
Changes in SAI of the two groups at different time points. a, b for letter labeling method, differences between time points within groups (*P* < 0.05). *represents the difference between the two groups at the same time (*P* < 0.05).

Before the intervention, SAI scores did not differ significantly between the two groups of primiparas (*P* > 0.05). A repeated-measures ANOVA revealed a significant main effect of time on SAI scores (*F* = 13.97, *P* < 0.001), but no significant main effect of group (*F* = 1.85, *P* = 0.177). A significant time-by-group interaction was observed (*F* = 2.88, *P* = 0.036), indicating that the trajectory of anxiety scores changed differently over time between the groups after the VR intervention. To decompose this interaction, simple effects analysis with adjustment was conducted. This analysis showed that SAI scores in the VR group remained stable, with no significant changes from baseline at any subsequent time point (all *P* > 0.05). In contrast, the control group showed significant increases in anxiety from baseline to both the preoperative (*P* = 0.000; 95% CI, −9.66 to −2.27) and intraoperative (*P* = 0.000; 95% CI, −12.22 to −3.89) assessments. Furthermore, between-group comparisons with Bonferroni correction confirmed that the VR group had significantly lower SAI scores than the control group at the preoperative time point (*P* = 0.028; 95% CI, −7.25 to −0.41; Table 2 and Figure 2).

**Table 2:** The results of repeated ANOVA for SAI.

Group	T0	T1	T2	T3	*F*-value	*P-*value	*F* for time (*P*-value)	*F* for group (*P*-value)	*F* for time group (*P*-value)
SAI							13.97 (0.000)	1.85 (0.177)	2.877 (0.036)
VR Group (*n* = 53)	40.00 ± 7.33	41.25 ± 8.75	43.04 ± 7.69	39.15 ± 9.47	2.161	0.097			
Control group (*n* = 54)	39.11 ± 9.89	45.07 ± 9.07	47.17 ± 13.54	38.96 ± 9.12	15.31	0.000			
*F-*value	0.29	4.935	3.744	0.011					
*P-*value	0.591	0.028	0.056	0.917					

Primiparas in the VR group reported a significantly lower delivery experience score (60.38 ± 17.73) than those in the control group (69.67 ± 19.37; *P* < 0.05; Table 3).

**Table 3:** The results of delivery experience, preoperative readiness, post-operative recovery and satisfaction.

Characteristics	VR group (*n* = 53) $\bar{\text{x}}$ ± *S*/*n*(%)	Control group(*n* = 54) $\bar{\text{x}}$ ±*S*/*n*(%)	*t/χ^2^*	*P-*value	Cohen's *d* (95% CI)
Delivery experience	60.38 ± 17.73	69.67 ± 19.37	−2.586	0.011	−0.5(−0.88 to −0.11)
Preoperative readiness	110.74 ± 8.15	104.56 ± 10.85	3.327	0.001	0.64 (0.25–1.03)
Satisfaction	117.51 ± 19.2	108.93 ± 20.07	2.26	0.026	0.44 (0.05–0.82)
Post-operative recovery	55.66 ± 16.51	52.06 ± 16.79	1.119	0.266	0.216 (−0.17 to 0.60)

### Secondary outcomes

Preoperative readiness and satisfaction were significantly higher in the VR group than in the control group (*P* < 0.05), whereas no significant between-group differences were observed in post-operative recovery outcomes (*P* >0.05; Table 3).

**Figure 3:**
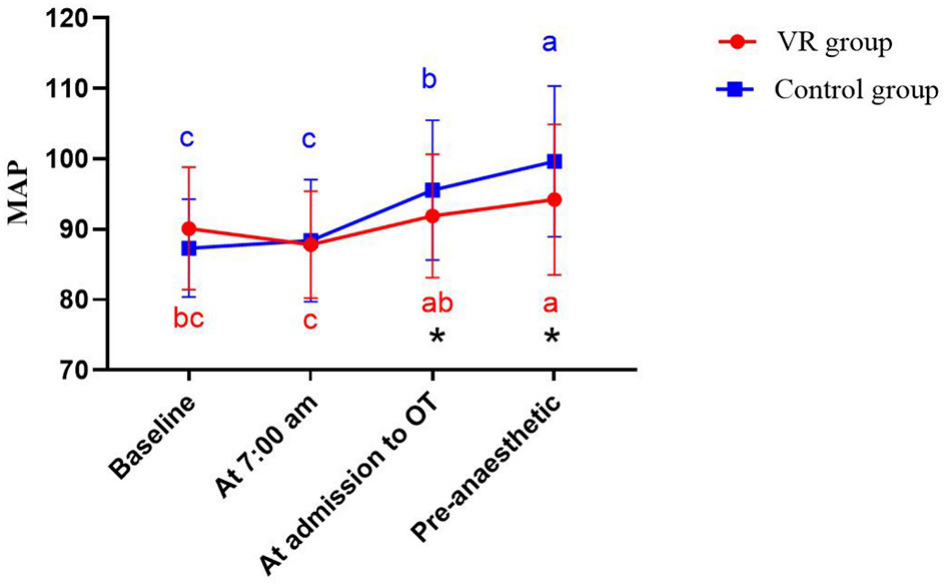
Changes in MAP of the two groups at different time points. a, b, c for letter labeling method, differences between time points within groups (*P* < 0.05). * represents the difference between the two groups at the same time (*P* < 0.05).

Prior to the intervention, MAP did not differ significantly between the two groups of primiparas (*P* > 0.05). A repeated-measures ANOVA revealed a significant main effect of time (*F* = 42.075, *P* < 0.001), a non-significant main effect of group (*F* = 1.624, *P* = 0.205), and a significant time-by-group interaction (*F* = 7.704, *P* < 0.001), suggesting that the VR intervention was associated with different patterns of MAP change over time between the groups. Simple effects analysis with Bonferroni adjustment, conducted to decompose this interaction, revealed that in the VR group, MAP values were significantly higher than baseline only at the pre-anesthesia time point (*P* = 0.021; 95% CI, 0.406 to 7.802). Conversely, in the control group, MAP values were significantly elevated compared to baseline at multiple time points: upon entering the operating room (*P* = 0.000; 95% CI, 4.824–11.645) and prior to anesthesia (*P* = 0.000; 95% CI, 8.630–15.975). Between-group comparisons with Bonferroni correction confirmed that the VR group had significantly lower MAP values than the control group both upon entering the operating room (*P* = 0.045; 95% CI, −7.259 to −0.078) and at the pre-anesthesia assessment (*P* = 0.010; 95% CI, −9.510 to −1.309; Table 4 and Figure 3).

**Table 4:** The results of repeated ANOVA for MAP, HR.

Group	Baseline	At 7:00 a.m.	At admission to OT	Pre-anesthetic	*F* (*P*-value)	*F* for time (*P*-value)	*F* for group (*P*-value)	*F* for time^*^group (*P*-value)
MAP						42.075 (0.000)	1.624 (0.205)	7.704 (0.000)
VR group (*n* = 53)	90.11 ± 8.69	87.81 ± 7.60	91.89 ± 8.75	94.22 ± 10.68	7.213 (0.000)	
Control group (*n* = 54)	87.33 ± 6.93	88.39 ± 8.67	95.56 ± 9.93	99.63 ± 10.71	32.895 (0.000)	
*F* (*P*-value)	3.351 (0.07)	0.137 (0.712)	4.104 (0.045)	6.844 (0.01)		
HR						10.330 (0.000)	0.115 (0.735)	2.227 (0.089)
VR group (*n* = 53)	88.04 ± 11.88	83.25 ± 6.07	88.30 ± 9.84	89.58 ± 11.64	5.665 (0.001)	
Control group (*n* = 54)	84.98 ± 11.91	81.61 ± 9.53	91.07 ± 11.65	93.50 ± 13.85	15.989 (0.000)	
*F* (*P*-value)	1.766 (0.187)	1.115 (0.293)	1.765 (0.187)	2.425 (0.122)		

^*^Represents the difference between the two groups at the same time point (*P* < 0.05).

**Figure 4:**
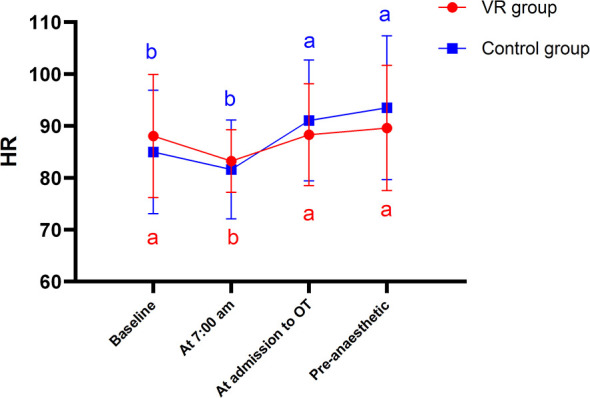
Changes in HR of the two groups at different time points. a, b for letter labeling method, differences between time points within groups (*P* < 0.05).

At baseline, HR did not differ significantly between the two groups of primiparas (*P* > 0.05). A repeated-measures ANOVA revealed a significant main effect of time (*F* = 10.330, *P* < 0.001), indicating that maternal HR changed significantly over time. However, neither the main effect of group (*F* = 0.115, *P* = 0.735) nor the time-by-group interaction (*F* = 2.227, *P* = 0.089) was statistically significant (Table 4 and Figure 4).

### Feedback on the VR experience

No primipara in the VR group reported adverse effects at baseline. During the intervention, one participant experienced transient mild dissociative symptoms, which resolved immediately upon removal of the VR device. Furthermore, the intervention was well-accepted, with 96% of primiparas expressing willingness to view the preoperative VR health promotion video.

## Discussion

Our findings suggest that VR may alleviate preoperative anxiety and appears to stabilize perioperative anxiety levels in primiparas, which is consistent with previous research (Ince and Karaman, 2024; Steinkraus et al., 2024; Xu, H et al., 2024; Xu, Y et al., 2024; Almedhesh et al., 2022). Simonov's (1990) cognitive theory of emotion emphasizes the pivotal role of cognition, proposing that emotions arise from the balance between information requirements and availability. According to this model, negative emotions result from an information deficit, positive emotions from a surplus, and emotional stability is achieved when information is sufficient to meet an individual's needs. This theoretical framework is highly relevant to primiparas, who, compared to multiparas, lack labor experience and consequently have a heightened need for procedural information. Preoperatively, they are particularly concerned about sensory experiences, including anesthetic discomfort and surgical pain intensity. In this study, primiparas in the VR group received detailed preoperative education about the surgical procedure and management of potential atypical sensations. From a cognitive perspective, fulfilling these informational needs reduces uncertainty, thereby mitigating preoperative anxiety and promoting perioperative emotional stability. Furthermore, the immersive nature of the VR environment facilitates controlled exposure to surgery-related stimuli. This process can promote desensitization to anxiety-provoking factors, thereby reducing fear before the actual experience. However, the null findings of Noben et al. (2019) regarding VR-based preoperative education can be explained by two key divergences in their methodology. First, their VR content emphasized procedural over sensory information, failing to prepare patients for discomfort. Second, the cohort included a significant share of multiparas, whose prior experience inherently buffers preoperative anxiety, thereby attenuating the observed pooled effect.

Our finding that VR improves the primiparas delivery experience converges with existing evidence on the positive impact of sensory information on emotional state and patient experience (Lu et al., 2022; Regan et al., 2019; Freeman et al., 2018). In this study, VR videos provided primiparas with preparatory sensory information about potential intraoperative sensations, including the nature, timing, and management of discomfort. This intervention enhanced their anticipation and perceived controllability over the procedure. Grounded in Antonovsky's theory of salutogenesis, this approach fosters a sense of coherence—comprehensibility, manageability, and meaningfulness—enabling individuals to combat stress and promote health. By preemptively building comprehensibility and controllability, the VR intervention equipped primiparas with the cognitive and psychological resources to better manage surgical stressors, thereby enhancing their overall delivery experience.

This study demonstrates that a VR-based intervention significantly enhanced preoperative readiness compared to conventional care. This effect is attributed to the multi-sensory, immersive nature of VR, which overcomes key limitations of traditional two-dimensional education (e.g., verbal instruction, text), which can be cognitively overwhelming and difficult to comprehend for patients without a medical background. By visually simulating preoperative procedures and potential sensory experiences within realistic scenarios, VR facilitated a deeper understanding of the surgery and equipped expectant mothers with strategies to manage adverse sensations. This dual enhancement of both cognitive comprehension and psychological coping mechanisms fostered comprehensive preoperative preparedness, a finding consistent with prior research (Chang et al., 2021).

Consistent with previous studies (Gerçeker et al., 2024; Corbaz et al., 2023), our results show that VR significantly improves maternal satisfaction. We posit that this effect is mediated through two primary pathways: first, through immersive engagement that fosters active participation in preoperative education; and second, through perceptual information support that prepares mothers for procedural sensations, thereby enhancing their perception of care quality. By mitigating preoperative negative emotions and improving perceived control, these mechanisms collectively optimize the entire perioperative experience, leading to higher overall satisfaction.

The stabilizing effect of VR on preoperative blood pressure observed in this study aligns with the findings of Almedhesh et al. (2022). This hemodynamic stability is likely attributable to the preoperative immersive exposure, which familiarized patients with the procedure, environment, and anesthetic protocols. This preparatory cognitive awareness mitigated the sympathetic nervous system's response to surgical stressors, leading to reduced catecholamine and adrenaline release. The consequent stabilization of cardiac contractility, coupled with lower preoperative anxiety that helped maintain endocrine equilibrium, collectively contributed to maintaining blood pressure within a consistent range. Contrast, the analysis for HR yielded a significant main effect of time but no significant group-by-time interaction. This discrepancy may be attributable to two interrelated considerations. Methodologically, as a physiological parameter with high intrinsic variability, instantaneous fluctuations in heart rate may have exerted a substantial influence on single-time-point or specific time-point measurements, potentially partially obscuring between-group differences. From a physiological perspective, sympathetic nervous system outputs exhibit effector specificity: they can modulate both cardiac output (via chronotropic and inotropic effects) and peripheral resistance (via vascular tone). We hypothesize that the VR intervention preferentially attenuated peripheral vascular sympathetic tone linked to anticipatory stress, thereby selectively reducing mean arterial pressure, while exerting a more limited effect on the direct cardiac sympathetic drive governing HR. This hypothesis of differential autonomic modulation warrants future validation using higher-resolution metrics, such as continuous heart rate variability analysis.

There was no significant difference in post-operative recovery indicators between the two groups. The reasons may be twofold. First, the VR intervention focused primarily on preoperative and intraoperative cognitive preparation, while post-operative rehabilitation guidance was limited to pain management, lacking systematic content such as early mobilization and nutritional support. Second, post-operative recovery is predominantly influenced by direct clinical factors including pain control, early ambulation capacity, surgical trauma, and complications. These factors depend on real-time medical interventions and individual physiological status, which are difficult to effectively modulate through a single, information-based preoperative health education session.

### Strengths and limitations

This study has several key strengths. First, the integration of perceptual information into the VR videos primed primiparas for potential intraoperative discomfort, detailing specific sensations and corresponding coping strategies. This approach enhanced the predictability and controllability of the surgical experience. Furthermore, the VR development process adhered to the guidelines set by the International VR Working Group, and the content was specifically tailored to the actual needs of primiparas, thereby maximizing therapeutic efficacy and improving the maternal experience.

This study has several limitations. Methodologically, the conspicuous nature of the VR intervention precluded participant blinding. As the primary outcomes were subjective patient-reported measures, this may have introduced expectation bias. Furthermore, the non-interactive nature of the VR content and the lack of assessment regarding participants' prior VR familiarity are potential confounding factors, as outcomes could be influenced by novelty effects or differing levels of technological acceptance. Additionally, although baseline anxiety was evaluated, participants with elevated anxiety symptoms were not excluded, nor was anxiety level incorporated as a stratification variable in the randomization process. This omission could have introduced variability in response to the intervention based on pre-existing anxiety, potentially confounding the interpretation of outcomes.

In terms of generalizability, our findings are derived from a single tertiary care center and a cohort exclusively comprising women undergoing elective cesarean delivery. Therefore, the results may not be applicable to the broader obstetric population, including those in different healthcare settings or those requiring emergency cesarean sections, who likely experience distinct psychosocial stressors due to the unpredictable nature of the procedure.

## Conclusion

In conclusion, this study demonstrates that VR therapy can effectively alleviate preoperative anxiety, enhance preparedness, improve the childbirth experience, and increase satisfaction among primiparas undergoing elective cesarean delivery. It thus demonstrates promise as a novel, non-pharmacological intervention for perioperative anxiety management. Future research should focus on developing VR content with interactive elements, conducting multi-center trials to validate its effects on intra- and post-operative mood, and expanding the study population to include women undergoing emergency cesarean sections to determine the broader applicability of this intervention.

## Data Availability

The original contributions presented in the study are included in the article/supplementary material, further inquiries can be directed to the corresponding author.
